# Effects and Microbiota Changes Following Oral Lyophilized Fecal Microbiota Transplantation Capsules in Canine with Chronic Enteropathy After Parvovirus Infection: Case Report

**DOI:** 10.3390/vetsci12090909

**Published:** 2025-09-18

**Authors:** Siyu Liu, Baihui Zhou, Lei Liu, Jialai Zhong, Xinyan Zhang, Wenting Jiang, Haifeng Liu, Ziyao Zhou, Guangneng Peng, Yalin Zhong, Kun Zhang, Zhijun Zhong

**Affiliations:** 1 Key Laboratory of Animal Disease and Human Health of Sichuan, College of Veterinary Medicine, Sichuan Agricultural University, Chengdu 611130, China; llsy6412024@163.com (S.L.); 410140017@163.com (H.L.); zzhou@sicau.edu.cn (Z.Z.); pgn.sicau@163.com (G.P.); 18190863559@163.com (Y.Z.); 15281297124@163.com (K.Z.); 2 Sichuan Anaerobic Biotechnology Co., Ltd., Chengdu 610200, China; zhoubaihui@scyysw.cn (B.Z.); liulei@scyysw.cn (L.L.); 3 Chengdu LifeMicro Technology Co., Ltd., Chengdu 610213, China; 4 Biogas Institute of Ministry of Agriculture and Rural Affairs, Chengdu 610041, China; zhongjialai@163.com; 5 College of Veterinary Medicine, Beijing University of Agriculture, Beijing 102206, China; xinyanzhang819@126.com; 6 College of Landscape Architecture, Sichuan Agricultural University, Chengdu 611130, China; wentingj1010@163.com

**Keywords:** fecal microbiota transplantation, diarrhea, dogs, intestinal microbiome, short-chain fatty acids

## Abstract

(1) Background: Chronic enteropathy (CE) in canines is associated with persistent microbiome dysbiosis, and conventional therapies (e.g., special diets, antimicrobials, and immunosuppressive drugs) are sometimes ineffective. Currently, fecal microbiota transplantation (FMT) has proven successful in treating CE in canines via invasive methods (e.g., enemas or endoscopy) or via oral frozen liquid capsules, which must be stored at −80 °C. However, due to the invasiveness of the administration methods and the storage constraints of the liquid capsules, FMT is not widely used in veterinary clinical practice. (2) Methods: The case of a four-year-old Siberian Husky with a three-year history of CE following canine parvovirus infection received lyophilized FMT capsules for thirty days. Stool samples were collected for metagenomic sequencing and quantification of fecal short-chain fatty acids (SCFAs), both pre- and post-FMT. Blood samples were analyzed using complete blood count (CBC) and biochemical testing. Ultrasound was used to assess the wall thickness of the stomach, duodenum, jejunum, and colon. (3) Results: Post-FMT, improvements in clinical outcomes were observed: fecal scores improved from 6 (unformed stools with mucus) to 2 (formed stool), and body weight increased by 8.3% (from 24.2 kg to 26.2 kg). Abnormal CBC and biochemical parameters were restored to reference ranges, including hematocrit (from 60.6% to 55.7%), hemoglobin (from 208 g/L to 190 g/L), creatinine (from 167 μmol/L to 121 μmol/L), and urea (from 11.9 mmol/L to 7.1 mmol/L). Ultrasound results showed that colonic wall thickness decreased from 0.23 ± 0.03 cm (pathological) to 0.18 ± 0.01 cm (physiological). Metagenomic analysis revealed that microbial richness (operational taxonomic units (OTUs) from 151 to 183) and diversity (Shannon and Simpson indices from 3.16 to 4.8 and from 0.87 to 0.94, respectively) all increased. The microbiota composition of the recipient exhibited a decline in the relative abundance of Firmicutes, falling from 99.84% to 35.62%, concomitant with an increase in Actinobacteria (from 0.08% to 4.78%), indicating a convergence toward a donor-like profile. Fecal SCFAs analysis revealed a 251.4% increase in propionate (from 0.0833 to 0.2929 mg/g) and elevated acetate (from 0.4425 to 0.4676 mg/g). These changes are functionally linked to enriched propanoate metabolism (Z = 0.89) in KEGG pathways. (4) Conclusions: Oral lyophilized FMT capsules resolved clinical signs of CE, enhanced microbial diversity and richness, and restored donor-like abundances of gut microbiota, particularly SCFA-producing taxa. Microbial restructuring increased microbial metabolite output, notably SCFA concentrations, and enriched functional metabolic pathways. Importantly, lyophilized FMT overcomes storage limitations and administration barriers, demonstrating its high clinical viability for treating canine CE.

## Simple Summary

This case report described the effects of an oral, lyophilized capsule fecal microbiota transplantation (FMT) treatment on a dog suffering from chronic diarrhea following a canine parvovirus infection. Remarkably, the recipient’s diarrhea resolved completely, and the stool consistency transitioned from a watery mucus-like state to a solid form post-FMT. Additionally, the recipient gained two kilograms in weight. We compared the fecal microbiomes of both the recipient and the donor, discussing our findings regarding bacterial engraftment in the recipient. Furthermore, an increase in metabolites (short-chain fatty acids) of the recipient was reported. Thus, oral lyophilized FMT capsules can be a promising treatment for dogs with chronic diarrhea that did not respond to conventional treatments.

## 1. Introduction

Chronic enteropathies (CE) are characterized by persistent gastrointestinal symptoms (e.g., vomiting and diarrhea) for ≥3 weeks, concurrent with intestinal dysbiosis [1]. Conventional CE therapies focus on specific diets (e.g., hydrolyzed protein diets) [2], antimicrobials (e.g., metronidazole or rifaximin) [3], and immunosuppressive drugs (e.g., prednisone) [4]. However, these can occasionally be ineffective, resulting in relapses [5,6]. Recent studies have shown that Fecal Microbiota Transplantation (FMT) can benefit patients by restoring intestinal microbial diversity and richness to a normal functional level [7,8,9]. FMT involves transferring the fecal microbiota of a healthy individual (donor) to a sick individual (recipient) to restore the recipient’s gut microbiota [10]. FMT first attracted attention due to its efficacy in treating Clostridium difficile infection (CDI) in humans [11]. In veterinary clinical practice, some recent reports have demonstrated the efficacy of FMT against diseases such as inflammatory bowel disease (IBD), acute hemorrhagic diarrhea syndrome (AHDS), and CDI in dogs [12,13,14,15]. A previous study showed that FMT can also significantly accelerate recovery from diarrhea and reduce hospitalization time when used as an adjunctive therapy in the treatment of canine parvovirus (CPV) [16].

Multiple studies demonstrate that treating diarrheic dogs with FMT promotes microbial convergence toward donor profiles and enhances microbial diversity [17,18]. In dogs with IBD, which is characterized by *Firmicutes*- and *Proteobacteria*-dominated dysbiosis, FMT markedly increases the relative abundance of *Fusobacteriota* and drives microbial convergence toward donor-like profiles [19]. In AHDS, FMT accelerates the restoration of gut microbial diversity and significantly increases the abundance of bacteria (such as *Faecalibacterium* and *Fusobacterium*) that produce short-chain fatty acids (SCFAs) [20,21], which play a dominant role in intestinal barrier repair and have anti-inflammatory effects [13]. Therefore, the restoration of essential SCFA-producing bacteria during the process of FMT could benefit canines with CE.

Compared to the widespread use of FMT in humans, reports on the application of FMT in canine patients are limited. To date, only a few clinical veterinarians have used this technique [22,23]. This may be because FMT administration in dogs was initially carried out via endoscopy or enema, both of which are limited by their inconvenient and invasive procedures [24,25]. However, recent studies have further demonstrated the efficacy of oral frozen liquid suspension capsules in treating diarrheic canines, with comparable results to endoscopy and enema administration [14,26,27,28]. However, these capsules must be stored at −80 °C, which is inconvenient for pet owners. In this case, we evaluated the therapeutic efficacy of oral lyophilized FMT capsules in a canine with CE by longitudinal monitoring of clinic-based parameters (fecal score and body weight) and changes in gut microbiota pre- and post-FMT.

## 2. Clinical Case Description

Patient History and Clinical Presentation. A four-year-old, unneutered female Siberian Husky (named Hua Sheng) presented with chronic diarrhea and vomiting lasting three years and was diagnosed with CE at the Teaching Animal Hospital of Sichuan Agricultural University based on the established criterion of persistent gastrointestinal signs exceeding three weeks [1]. According to the owner’s description, Hua Sheng was infected with canine parvovirus at one year of age. Following the resolution of the infection, the dog exhibited symptoms of chronic diarrhea, which have persisted to the present. Clinical manifestations included unformed stools with mucoid discharge and intermittent vomiting (two or three times per month). The following therapeutic interventions were administered: oral anthelmintic therapy (praziquantel), antimicrobial treatment (azithromycin), and systemic anti-inflammatory injections. However, only transient symptomatic improvement was observed, with no sustained resolution of clinical signs.

FMT Capsule Preparation. FMT capsules were prepared from fecal material donated by a 19-month-old male beagle (3.5 kg body weight), which was housed at an experimental breeding facility in Dujiangyan City, Sichuan Province. The facility implemented strict biosafety management practices involving daily disinfection protocols and restricted access. The donor was fed a standardized, commercially formulated diet that complied with the Chinese national standard GB 14924.1-2001 [29]. Prior to fecal sample collection, the donor underwent comprehensive health assessments: (1) complete blood count (CBC) and serum biochemistry profiles within the normal reference ranges; (2) an absence of parasites (Cryptosporidium, Giardia and Tritrichomonas foetus) in fecal examinations; and (3) no pathogens were detected in the screening for the three common diarrhea viruses: canine parvovirus, distemper virus and coronavirus.

**Figure 1:**
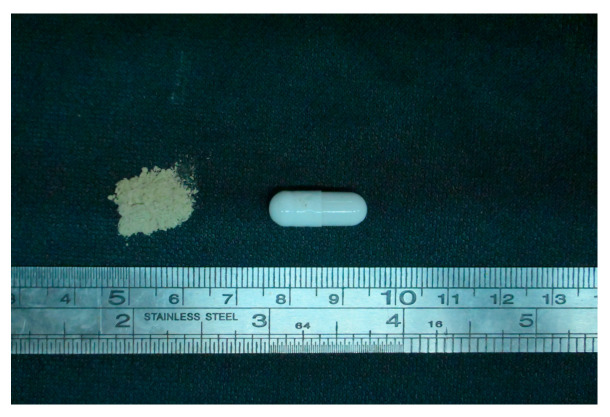
Oral lyophilized fecal microbiota transplantation capsules.

The FMT capsules were manufactured in accordance with our previously established patent (CN118638635A) [30]. In brief, 100 g of fresh donor stool (wet weight) was homogenized with sterile physiological saline using a paddle homogenizer. The resulting suspension was then filtered sequentially through graded meshes (30 μm; 40 μm; 50 μm) to remove particulate matter. The filtrate was then centrifuged at 4000× *g* for 10 min at 4 °C, and the resulting pellet was collected as the bacterial pellet. This pellet was blended with lyoprotectants (6% trehalose, 20% sucrose, 1% PEG-3350, 1% glycerol, 1% sodium glutamate, 2.5% sodium ascorbate, balance sterile water) at a mass ratio of 1:1.5 and emulsified to homogeneity. The mixture was then lyophilized for 48 h to produce microbial powder, which was pulverized and encapsulated in size 2 enteric-coated capsules (see Figure 1). The final products were stored at 2–8 °C. The total viable bacterial cell count in the FMT capsules is 4.11 × 10^8^ CFU/g. Each capsule contained 100 mg of lyophilized powder, with a viable count of 4.11 × 10^7^ CFU per capsule. The post-lyophilization survival rate exceeded 80%, and viability remained above 80% after 30 days of storage at 2–8 °C.

FMT Treatment Protocol. Prior to the present case, a pilot investigation by our team demonstrated that canines weighing kilograms or more who were given four capsules daily achieved better outcomes than those given two capsules daily. It is important to emphasize that this preliminary dosing strategy was exploratory and was not derived from an established or optimized protocol. Based on these findings, Hua Sheng was given four capsules orally each morning on an empty stomach for 30 days. CBC, biochemical analysis, C-reactive protein (CRP), and abdominal ultrasonography were tested at the Teaching Animal Hospital of Sichuan Agricultural University on Days 0, 15, and 30. Before FMT treatment, the recipient underwent a four-week drug-free washout period, which included all antimicrobials, anti-inflammatory drugs, and probiotics. During both the treatment and follow-up phases, the recipient was fed a consistent home-cooked diet (rabbit meat 290 g, potato 40 g, broccoli 25 g, oatmeal 25 g, bok choy 20 g, and flaxseed oil 2 g) and was prohibited from using any probiotics, antibiotics, or other gastrointestinal medications. CBC analysis was performed on venous blood samples (1 mL) and processed using an IDEXX ProCyte Dx analyzer to quantify erythrocyte, leukocyte, and platelet indices. Biochemical profiling was conducted on plasma obtained from heparinized whole blood (2 mL centrifuged at 4007× *g* for 3 min), with immediate quantification of alanine aminotransferase (ALT), alkaline phosphatase (ALP), blood urea nitrogen (BUN), and CRP levels via an IDEXX Catalyst One system. Ultrasonography was performed by a veterinary radiologist using a high-resolution system (GE Logiq E10 with an ML6-15-D linear probe at 15 MHz). Triplicate measurements were taken to assess the thickness of the gastric wall at the greater curvature (in a fasted state), the duodenal mucosa-submucosa complex in the proximal descending segment, and the jejunal and colonic walls within fluid-filled, non-peristaltic loops. According to the literature, the reference values for the duodenum, jejunum, and colon are 0.3–0.55 cm, 0.24–0.48 cm, and 0.11–0.19 cm, respectively [31]. Fecal samples collected during spontaneous defecation from dogs were cryopreserved at −80 °C for metagenomic and short-chain fatty acids (SCFAs) analysis. Daily fecal morphology was assessed using the validated Bristol Stool Form Scale (scale 1–7) [32].

FMT Efficacy Assessment via SCFAs Quantification and Metagenomic Profiling. The concentration of SCFAs was quantified using an Agilent 7890A gas chromatograph (Agilent Technologies, Santa Clara, CA, USA) according to the method described by Park HE et al. [33]. The stool samples were homogenized by transferring an accurate weight of 0.5 g of the sample to a 2 mL centrifuge tube. Add 1.2 mL of ultrapure water (the volume should be proportional to the sample mass), vortex to mix, then leave to stand for 5 min at room temperature. Centrifuge the suspension at 12,272× *g* for 15 min to precipitate insoluble debris. Then, 1 mL of the supernatant was mixed with 0.2 mL of 25% (*w*/*v*) metaphosphoric acid solution and 23.3 μL of 210 mmol/L crotonic acid solution (the volumes were adjusted proportionally to maintain the same ratio), incubated at 4 °C for 30 min, and then centrifuged at 4007× *g* for 10 min. Then, 0.3 mL of the clarified supernatant was diluted with 0.9 mL of chromatography-grade methanol (1:3 dilution ratio, adjusted to scale), vortexed, and centrifuged at 4007× *g* for 5 min. The final supernatant was filtered through a 0.22 μm nylon membrane and loaded into a gas chromatography vial for analysis. The conditions were as follows: HP-FFAP capillary column (30 m × 0.32 mm × 0.32 μm), using a high-purity nitrogen carrier gas at a flow rate of 2.2 mL/min. The inlet temperature was 250 °C. The oven programme started at 60 °C (held for 1 min), increased to 170 °C at a rate of 10 °C/min, and then to 212 °C at a rate of 8 °C/min (held for 2 min).

Following sample quality control (QC), 500 ng of metagenomic DNA was fragmented using the Covaris E220 system (Covaris, Brighton, UK). The fragmented DNA was then size-selected to a range of 300–700 bp using magnetic bead-based size selection. The selected DNA fragments underwent end repair and were ligated to indexed adapters. The ligation products were then amplified via PCR, hybridized with exon-specific probes, and captured using streptavidin-coated beads. The captured DNA underwent a second round of PCR amplification and was circularized to generate a single-stranded circular (ssCir) library. This ssCir library was then amplified using rolling circle amplification (RCA) to produce DNA nanoballs (DNBs). Finally, the DNBs were loaded onto a flow cell and sequenced using the DNBSEQ platform.

Metagenomic assembly and analysis. Metagenomic analyses began with a quality assessment of the raw sequencing data using FastQC (v0.12.1). Trimmomatic (v0.39) was then used for adapter and quality trimming with the parameters SLIDINGWINDOW:4:15 and MINLEN:99 to retain high-quality reads. Host-derived sequences were removed by aligning the reads to the reference genome of Canis lupus familiaris (GCF_011100685.1) using Bowtie2 (v2.5.4). The filtered reads were assembled de novo into contigs (≥500 bp) using MEGAHIT (v1.2.9) with iterative k-mer optimization (k-min: 27, k-max: 127). Open reading frames (ORFs) were predicted using Prodigal (v2.6.3) in metagenomic mode (-p meta). Functional annotation was performed against the KEGG database (KOfam release 2024) with stringent thresholds (E-value < 1 × 10^−5^ and coverage >70%). The ORFs were further annotated against the eggNOG 6.0 database (covering 5635 bacterial and archaeal taxa) using eggNOG-mapper v2.1.6 in Diamond mode to assign Clusters of Orthologous Groups (COG) categories. MetaPhlAn4 (v4.0.2) was used to quantify species-level relative abundance, and absolute abundance was derived by multiplying relative abundance by sample-specific, quality-controlled read counts. Alpha diversity was assessed using Pielou’s evenness (J = H′/ln(S)), Shannon entropy (H = −∑pi*ln(pi)), and Simpson index (D = 1 − ∑(ni/N)^2^). Beta diversity was computed using QIIME 2 (v2024.2). Visualizations were generated using GraphPad Prism (v10.0) and TBtools (v2.0).

## 3. Results

### 3.1. Clinical Outcomes

#### 3.1.1. Hematological Parameters

Prior to FMT, CBC analysis indicated that most hematological parameters were within the normal reference ranges (Table 1). Only four parameters were abnormal: hemoglobin (HGB, 208 g/L), hematocrit (HCT, 60.6%), mean corpuscular volume (MCV, 74.1 fL), and mean corpuscular hemoglobin (MCH, 25.4 pg). Post-FMT, all indicators were within the reference ranges, with all of the previously abnormal parameters (HGB, HCT, MCV, and MCH) returning to normal levels. Pre-FMT, biochemical profiling revealed that all biochemical profiling and CRP levels remained within the reference ranges, except for creatinine (CREA, 167.0 μmol/L) and urea (UREA, 11.9 mmol/L) (Table 2). Post-FMT, both CREA (121.0 μmol/L) and UREA (7.1 mmol/L) declined, falling within the reference ranges. Overall, FMT effectively restored all abnormal hematological and biochemical parameters to physiological levels.

#### 3.1.2. Fecal Score and Body Weight Recovery

**Figure 2:**
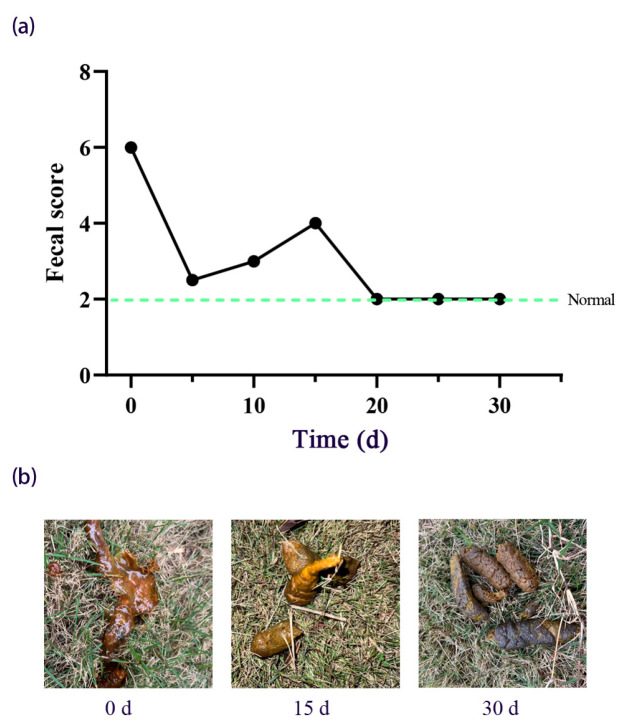
Restoration of fecal morphology following FMT therapy in the recipient dog. (**a**) Dynamic changes in fecal scores assessed by modified Bristol Stool Form Scale (range 1–7, dashed green line: physiological threshold at score 2). (**b**) Fecal samples at critical time points: T0 (unformed stools with mucus, score 6), T15 (semi-formed stools with mucus, score 4), and T30 (formed stools, score 2).

**Figure 3:**
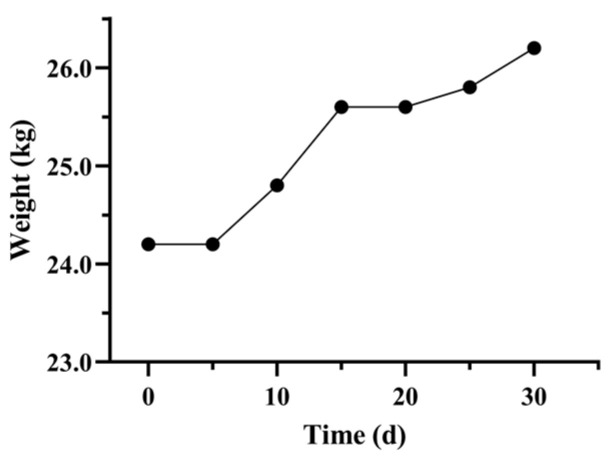
Body weight changes in the recipient treated with 30 days of FMT.

As Figure 2 shows, pre-FMT, the fecal score was 6 (unformed feces with mucus). From T0 to T5 post-FMT, the fecal score decreased to 2.5. From T5 to T20 post-FMT, there was a fluctuating downward trend (scores 2–4), characterized by intermittent soft stools and minimal mucus. Between T20 and T30 post-FMT, fecal score stabilized at 2 (formed feces). Figure 3 shows the details of the change in body weight during FMT. From T0 to T5, body weight remained at 24.2 kg. From T5 to T15, there was a moderate increase in body weight (from 24.2 to 25.6 kg). By T30, the body weight had increased to 26.2 kg. Our results showed that the resolution of diarrhea occurred alongside sustained weight restoration.

#### 3.1.3. Gastrointestinal Morphology Evaluation

**Figure 4:**
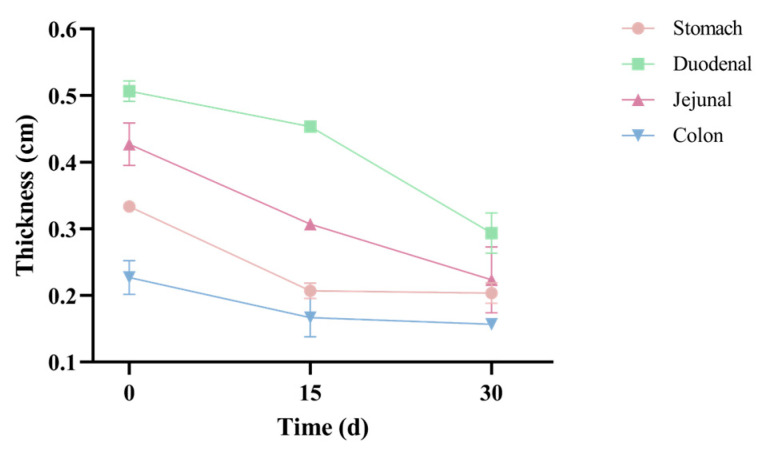
Ultrasonographic measurement of gastrointestinal wall thickness during FMT treatment. Line graphs depict changes in wall thickness (cm) of the stomach (pink), duodenum (green), jejunum (purple), and colon (blue) at baseline (T0) and T15 and T30 post-FMT administration in the recipient dog. Data points represent mean thickness ± SD (error bars).

As Figure 4 shows, pre-FMT, the wall thicknesses of the stomach (0.33 ± 0.01 cm), duodenum (0.51 ± 0.02 cm), and jejunum (0.43 ± 0.03 cm) were all within the reference ranges (duodenum: 0.3–0.55 cm; jejunum: 0.24–0.48 cm). Only the colonic wall thickness (0.23 ± 0.03 cm) was outside the reference range (0.11–0.19 cm). Post-FMT, colonic wall thickness (0.16 ± 0.01 cm) returned to the normal range. Our results showed that FMT effectively restored pathological colonic wall thickness to standard physiological levels.

### 3.2. Effects of FMT on Fecal Microbial Communities

#### 3.2.1. Microbial Composition

**Figure 5:**
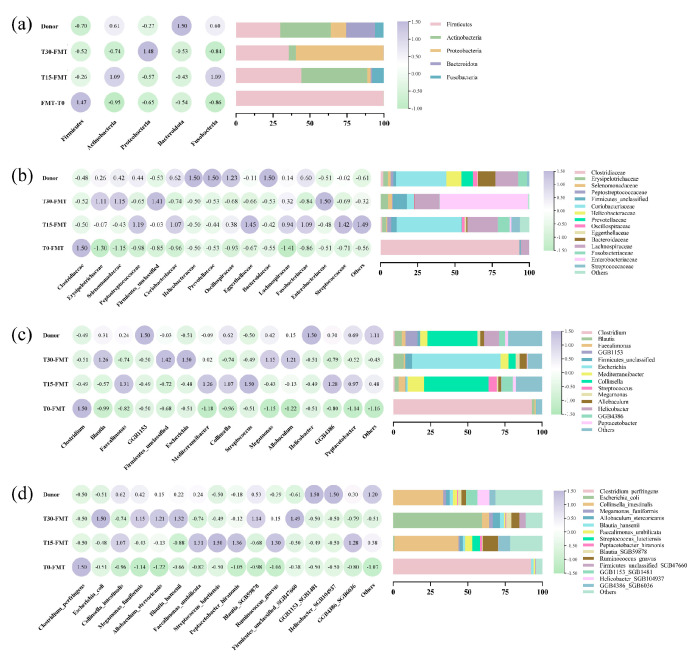
Analysis of intestinal microbial composition. (**a**) Left panel displays a heatmap of phylum-level abundances (graded blue to purple, indicating low-to-high relative abundance). Right panel shows a horizontal stacked bar chart of phylum composition. (**b**) Left panel displays a heatmap of family-level abundances. Right panel shows a horizontal stacked bar chart of the top fifteen most abundant taxa of family composition. (**c**) Left panel displays a heatmap of genus-level abundances. Right panel shows a horizontal stacked bar chart of the top fourteen most abundant taxa of genus composition. (**d**) Left panel displays a heatmap of species-level abundances. Right panel shows a horizontal stacked bar chart of the top fifteen most abundant taxa of species composition. All heatmaps and horizontal stacked bar charts include donor (baseline) and FMT timepoints (T10, T15, T30).

Figure 5 shows the dynamic shifts in the recipient of gut microbiota during FMT at T0, T15, and T30. The relative abundances of all microbiota composition data are provided in Supplementary Table S1. At the phylum level (Figure 5a), the donor microbiota was dominated by *Firmicutes* (29.84%) and *Actinobacteria* (34.10%). By contrast, the recipient’s microbiota pre-FMT exhibited extreme *Firmicutes* dominance (99.84%), which declined to 35.62% at T30 post-FMT. This was concurrent with an increase in *Actinobacteria* (from 0.08% to 4.78%). At the family level (Figure 5b), post-FMT, the relative abundances of *Erysipelotrichaceae* (from 0.06% to 4.96%), *Selenomonadaceae* (from 0.00% to 2.70%), *unclassified Firmicutes* (from 0.67% to 9.30%), *Coriobacteriaceae* (from 0.02% to 9.30%), and *Lachnospiraceae* (from 5.54% to 16.55%) increased, while the relative abundance of *Clostridiaceae* decreased from 93.22% to 0.23%, eventually approaching donor-like abundances (3.23%, 1.84%,1.87%, 33.69%, 15.44% and 1.87%, respectively). At the species level, the relative abundances of *Megamonas funiformis* (from 0% to 2.7%), *Allobaculum stercoricanis* (from 0% to 4.64%), *Blautia hansenii* (from 1.68% to 2.64%), *Ruminococcus gnavus* (from 0.06% to 5.31%), and *Blautia_SGB59878* (from 0.00% to 3.47%) increased and converged toward donor-like abundances (1.84%, 2.62%, 2.10%, 2.94%, and 2.47%, respectively). Notably, genus and species analysis (Figure 5c,d) revealed a taxonomic shift in the recipient’s dominant microbiota: *Clostridium perfringens* was the most prevalent species pre-FMT (92.41%), while *Collinsella intestinalis* was the dominant species at T15 (42.98%), with *Escherichia coli* becoming dominant at T30 post-FMT (59.42%). Our results showed that the relative abundance of the recipient’s microbiota post-FMT converged toward donor-like abundances.

**Figure 6:**
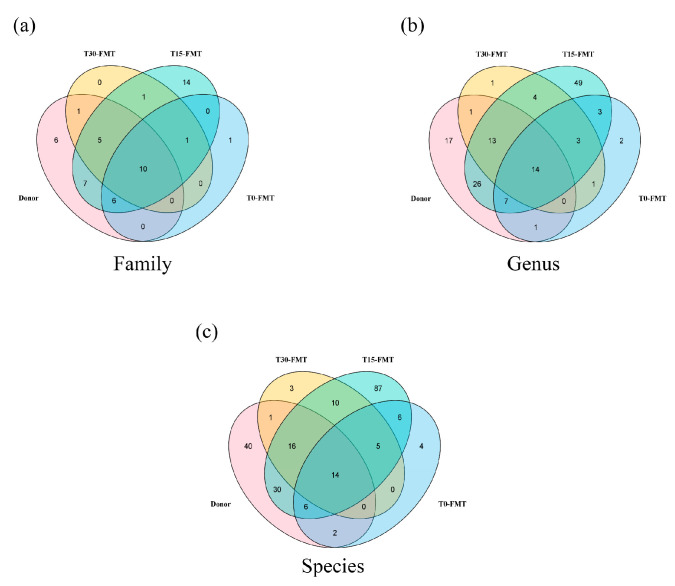
Venn diagrams showcasing the unique and shared microbiota among the donor at baseline and the recipient at T0, T15, and T30. In the Venn diagram, the number within the overlapping circles denotes the count of the shared OTUs. (**a**) Venn diagram on family level. (**b**) Venn diagram on genus level. (**c**) Venn diagram on species level.

Alterations in microbiome composition were further manifested by the overlap of operational taxonomic units (OTUs) between the donor and recipient. At the family level (Figure 6a), a total of 52 OTUs were observed. Ten OTUs were shared by all the samples, and the number of unique OTUs for the recipient at T15 (14) was higher than in other samples. At the genus level (Figure 6b), 14 OTUs were shared by all the samples. The number of unique OTUs for the donor at baseline and for the recipient at T0, T15, and T30 was 17, 1, 49, and 2, respectively. At the species level (Figure 6c), the number of shared OTUs between the recipient and the donor was just 14; most OTUs were unique, with 87 unique OTUs observed in the recipient at T15. Notably, the recipient at T15 exhibited maximal taxonomic overlap with the donor at family, genus, and species levels.

#### 3.2.2. Diversity Analysis

**Figure 7:**
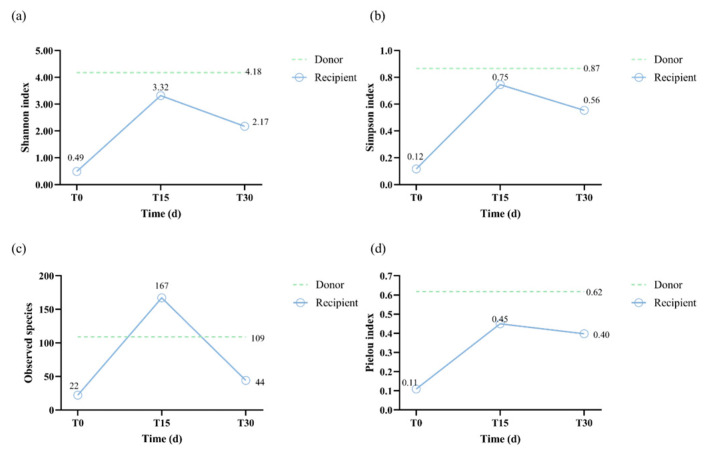
Alpha diversity analysis: (**a**) the values of the Shannon index; (**b**) the values of the Simpson index; (**c**) the values of the observed species; (**d**) the values of the Pielou index. The values were shown as a blue polyline for the recipient, and the reference values for the donor are shown in dashed green.

As shown in Figure 7a,b, pre-FMT, the recipient’s Shannon and Simpson indices (0.49 and 0.12, respectively) were lower than the donor’s (4.18 and 0.87, respectively). At T15 post-FMT, both Shannon and Simpson indices demonstrated a peak in the recipient (3.32 and 0.75, respectively), approaching donor-like levels. Post-FMT, Shannon and Simpson indices showed a modest decline (2.17 and 0.56, respectively), yet remained elevated compared to pre-FMT levels. Figure 6c shows a similar trend in the observed species of the recipient during FMT. Pre-FMT, the recipient’s microbiota exhibited low species richness (22 OTUs), surging to 167 OTUs at T15 and exceeding donor-like levels (109 OTUs). Post-FMT, richness declined to 44 OTUs, though this remained higher than pre-FMT levels. From T0 to T30, the Pielou index showed a fluctuating upward trend, from 0.11 to 0.40, approaching the donor-like level (0.62). These results demonstrate that FMT initially led to a surge in microbial diversity and richness, followed by partial stabilization, while achieving sustained improvement in community evenness.

**Figure 8:**
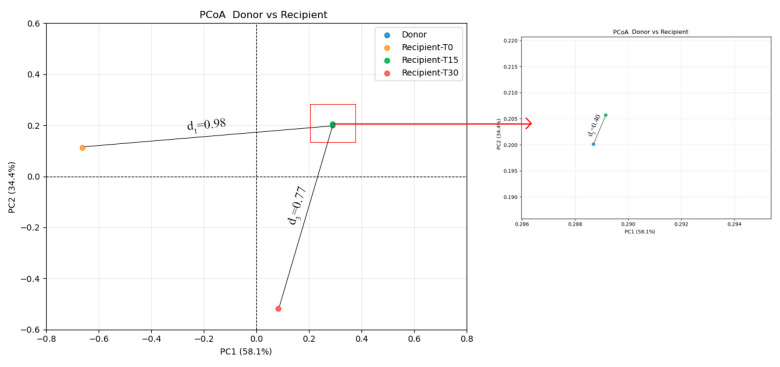
Principal coordinate analysis (PCoA) of the beta diversity of gut microbiota. The left panel shows the full-scale ordination of all samples (PC1 = 58.1%, PC2 = 34.4%; cumulative variance = 92.5%). Solid lines indicate the distances d1 (donor vs. pre-FMT recipient) and d3 (donor vs. T30 recipient). The right panel provides an enlarged view of the red boxed region, revealing clustering of the donor and recipient samples at T15. The distance d2 (donor vs. T15 recipient) is measured under magnification.

To further investigate differences in microbial structure between recipients and donors, beta diversity analysis was performed at T0, T15, and T30. Principal coordinate analysis (PCoA) showed that the first two principal coordinates (PC1 and PC2) together explained 92.50% of the beta diversity variance (Figure 8). Bray–Curtis distances showed that d_3_ (0.77; donor vs. post-FMT recipient) and d_2_ (0.40; donor vs. T15 post-FMT recipient) were smaller than d_1_ (0.98; donor vs. pre-FMT recipient). These results suggest that FMT effectively restructured the composition of the recipient’s gut microbiome, driving it toward a donor-like profile.

### 3.3. SCFAs and Metagenome Functions

To evaluate the impact of FMT on key SCFAs, we quantified the concentrations of acetate (AA), propionate (PA), and butyrate (BA) at T0 and T30. Pre-FMT, the concentrations of AA, PA, and BA were 0.4425 mg/g, 0.0833 mg/g, and 0.2971 mg/g, respectively. Post-FMT, AA and PA increased to 0.4676 mg/g (+5.7%) and 0.2929 mg/g (+251.4%), respectively, whereas BA decreased by 36% to 0.1900 mg/g.

**Figure 9:**
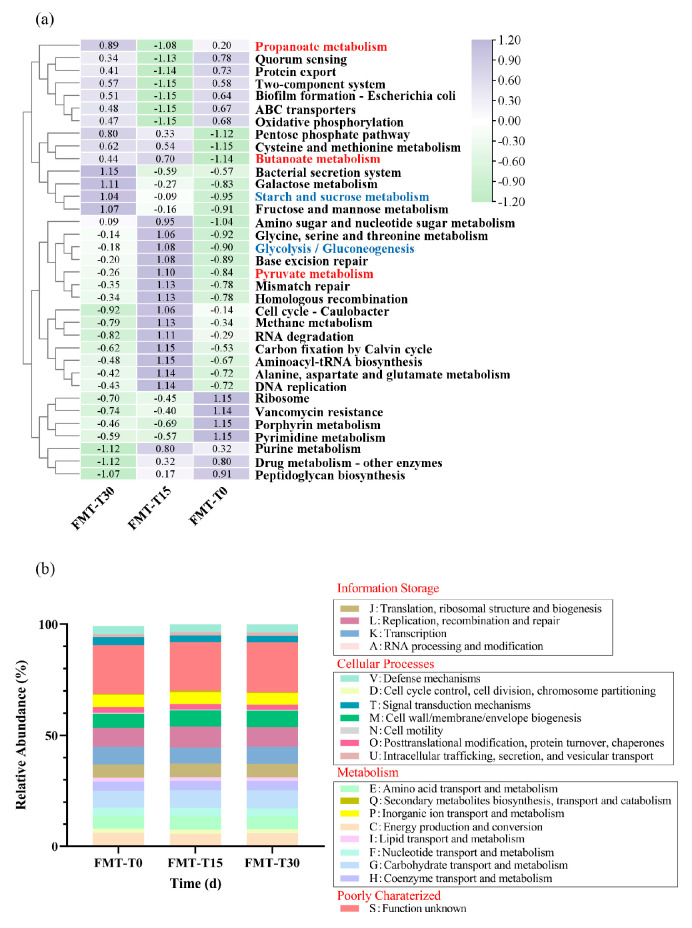
Functional prediction of gut microbiota via metagenomics. (**a**) Heatmap of KEGG pathways significantly altered post-FMT. Pathways directly and indirectly associated with fatty acid metabolism are highlighted in red and blue text, respectively. (**b**) Stacked bar plot of COG functional category abundances across timepoints.

To further investigate functional alterations in the metagenome associated with SCFAs metabolism, we performed Kyoto Encyclopedia of Genes and Genomes (KEGG) enrichment analysis and Cluster of Orthologous Groups of proteins (COG) functional analysis. As shown in Figure 9a, KEGG identified the top 35 significant pathways. The relative abundances of all KEGG pathways are provided in Supplementary Table S2. During FMT, direct pathways related to fatty acid metabolism showed significant upregulation, including propionate metabolism (z-score ranging from 0.20 to 0.89), pyruvate metabolism (z-score ranging from −0.84 to −0.26), and butanoate metabolism (z-score ranging from −1.14 to 0.44). Additionally, post-FMT, indirect pathways of fatty acid metabolism also showed upregulation, such as starch and sucrose metabolism (z-score ranging from −0.95 to 1.04) and glycolysis/glucogenesis (z-score ranging from −0.84 to −0.18). COG functional analysis (Figure 9b) revealed that the top 5 abundant pathways were L [Cell cycle control], K [Transcription], G [Carbohydrate transport and metabolism], M [Cell wall/membrane biogenesis], and C [Energy production and conversion]. Post-FMT, the dominant pathways shifted toward G, J [Translation, ribosomal structure and biogenesis], K, L, and M, with the relative abundances of pathways G, L, and M increasing, and those of C, J, and K declining. The relative abundances of all COG categories are provided in Supplementary Table S3.

## 4. Discussion

Many studies demonstrate that microbial dysbiosis consistently accompanies CE, subsequently inducing chronic diarrhea [34,35,36]. The ‘CE-dysbiosis-diarrhea’ cycle constitutes a self-perpetuating pathological loop. Restoring the gut microbiota is a vital intervention for breaking this cycle. A previous study reported that those who survive CPV infection have a significantly higher risk (odds ratio = 5.33) of developing chronic gastrointestinal disease [37]. In this case, we successfully treated a canine with CE that had persisted for three years following canine parvovirus infection using oral lyophilized FMT capsules, with no recurrence observed over a 6-month follow-up period (fecal scores consistently maintained at 2).

Fecal score and body weight are clinically relevant and visually intuitive indicators of diarrheal recovery [38]. In the present study, the fecal score decreased from 6 (unformed, mucus-covered stool) to 2 (formed, healthy stool), with no recurrence observed over a 6-month follow-up period. A previous study also showed that fecal scores normalized to 2 in dogs treated with FMT [15]. Body weight increased from 24.2 to 26.2 kg, and remained 26.3–26.8 kg over a 6-month follow-up period. Several studies have demonstrated that FMT enhances the body weight of patients [39,40]. Change in other laboratory indicators (hematological indices and ultrasonographic intestinal wall thickness) suggested that the FMT intervention achieved clinically relevant improvements.

The therapeutic benefit of FMT lies in its capacity to restore intestinal microbial diversity and richness, thereby re-establishing microecological balance in recipients [7,41,42]. Alpha and beta diversity were commonly used to reflect microbial diversity and richness. In our present study, the alpha diversity indices (Pielou, Shannon, and Simpson; see Figure 6a,b) revealed an increase in microbial diversity post-FMT (0.40, 2.17, and 0.56, respectively) compared to the pre-FMT values (0.11, 0.49, and 0.12). This suggests that FMT can reverse the reduction in gut microbiota richness induced by diarrhea. We observed that the Shannon and Simpson indices exhibited an initial increase followed by a subsequent decline during the FMT, which was consistent with a previous study [43]. Transient peaks in the Shannon and Simpson indices at T15 (see Figure 6b) correspond with the established engraftment dynamic whereby the initial introduction of donor microbiota leads to an increase in diversity, followed by selective retention of engrafted taxa [43,44]. Regarding beta diversity, PCoA results showed that the post-FMT microbiota were closer to the donor profile than the pre-FMT microbiota, indicating progressive convergence toward donor-like communities. Similarly, Goll et al. recently reported that FMT responders had a tendency to converge toward the donor microbiome profile after FMT, whereas this trend was less evident in non-responders [45]. This shift is consistent with established predictors of successful FMT, whereby increased alpha diversity and donor-like beta diversity are critical determinants of therapeutic efficacy [46]. The results of the beta diversity analysis were consistent with the observed changes in microbiota structure. In this case, the main shift occurred at the phylum level, reflecting a dynamic microbial restructuring process following FMT. Pre-FMT, *Firmicutes* (especially *C. perfringens*) was the most dominant phylum; at T15, *Actinobacteria* (especially *C. intestinalis*) became dominant; and post-FMT *Proteobacteria* (especially *E. coli*) became dominant at T30. This pattern of microbial succession may be driven by competitive exclusion, in which *C. perfringens* was initially replaced by *C. intestinalis* at T15, followed by *E. coli* at T30 [47,48]. Specifically, the expansion of E. coli at T30 occurred following a substantial decline in *C. intestinalis* (from 42.98% at T15 to 4.73% at T30), suggesting that competitive exclusion created a temporary niche opportunity that allowed *E. coli* to bloom. Thus, the transient dominance of E. coli can be interpreted as part of the ecological restructuring process, ultimately contributing to the restoration of microbial balance. These findings align with a previous study documenting the role of competitive exclusion in human CDI, whereby introduction of non-toxigenic strains reduces CDI recurrence [49]. Concurrently, FMT enriched beneficial microbial families (e.g., *Lachnospiraceae*, *Ruminococcaceae*, and *Erysipelotrichaceae*), which have the ability to produce SCFAs and induce anti-inflammatory Treg responses [50,51]. Specifically, the SCFA-producing genera *Blautia*, *Peptacetobacter*, and *Ruminococcus* are key microbial groups for canine intestinal health and play an important role in digesting complex carbohydrates, enhancing immunologic function, and preventing pathogenetic invasion [52,53]. In our case, the levels of *Blautia*, *Peptacetobacter,* and *Ruminococcus* microbial groups increased variably post-FMT and approached donor-like levels, suggesting that these microbial groups may have played a vital role in repairing the intestinal microbiota community and alleviating CE symptoms. In summary, FMT has been shown to disrupt the dysbiosis-inflammation cycle, thereby facilitating the restoration of microbial homeostasis. This restoration enhances microbial diversity and richness and enriches SCFA-producing microbes.

In addition to increasing the diversity and richness of the microbiota following FMT, bacterial metabolites such as SCFAs benefit the recipient by enhancing the intestinal barrier and energy metabolism [54,55]. We analyzed the three main SCFAs (acetate, butyrate, and propionate) at T0 and T30 to evaluate the restoration of metabolic activity in microbiota. Our results showed that the concentrations of two SCFAs (acetate and propionate) increased (from 0.4425 to 0.4676 mg/g and from 0.0833 to 0.2929 mg/g, respectively). Acetate and propionate positively affect glycolysis and propionate metabolism [56]. KEGG enrichment analysis revealed that glycolysis and propionate metabolism were significantly increased, suggesting that elevated acetate and propionate levels enhance carbohydrate metabolism and provide additional energy for the body [56]. A similar conclusion was obtained in a previous study, which found that propionate levels were increased significantly (p = 0.025) post-FMT [57]. Butyrate has been shown to possess anti-inflammatory properties, thereby promoting intestinal barrier function [58,59]. However, our case report revealed a 36% decrease in butyrate concentration (from 0.2971 mg/g to 0.1900 mg/g). A previous study demonstrated that the phylum Firmicutes (particularly the species *C. perfringens*) has the capacity to produce butyrate [60]. In our case, we found a decline in *C. perfringens* (from 92.41% to 0.03%) abundance. We therefore hypothesize that the decreased butyrate concentration may be related to the reduction of *C. perfringens* abundance. It is important to note that this is a correlative observation based on a single case, and a causative relationship cannot be established here. Although the concentration of butyrate declined, enrichment of the KEGG pathway in butyrate metabolism post-FMT indicates that the recipient’s intestinal microbes may have acquired the capacity to synthesize butyrate. Thus, we speculate that the sharp reduction in *C. perfringens* may not be sufficient to rapidly restore butyric acid concentrations within the short term. Thus, the concentration of butyrate may require a longer period to manifest. This interpretation remains speculative and warrants further investigation in larger cases to validate the underlying mechanisms.

The limitations of this case report are as follows: (1) the limited sample size; larger case studies should be validated; (2) use of a single donor; multi-donor protocols should be prioritized to ensure broader applicability; (3) short follow-up period (30 days), extended monitoring is needed to evaluate long-term efficacy and safety. Nevertheless, the observed clinical recovery (no diarrhea recurrence at 6 months) and microbial restoration support the potential of oral lyophilized FMT capsules, despite these limitations.

## 5. Conclusions

In this case report, we successfully treated a canine with CE that had persisted for three years following canine parvovirus infection using oral lyophilized FMT capsules. Clinical improvements were observed, including the cessation of diarrhea with no recurrence over a 6-month follow-up period, as well as body weight gain. Concurrently, Microbial analysis revealed the enhanced diversity and richness of the gut microbiota following FMT, along with an increase in SCFA-producing bacteria and restoration of SCFAs-related metabolic pathways. This case provides a proof-of-concept example that oral lyophilized FMT may supply a therapeutic alternative for chronic diarrhea in dogs.

## Figures and Tables

**Table 1:** CBC values at baseline (T0) and post-FMT time points (T15 and T30) in the recipient.

Indicators	Unit	FMT-T0	FMT-T15	FMT-T30	Reference
HGB	g/L	208	192	190	110–190
HCT	%	60.6	55.1	55.7	39.0–56.0
WBC	10^9^/L	11.1	8.4	10.5	6.0–17.0
Lymph	10^9^/L	1.8	2.0	2.3	0.8–5.1
Mon	10^9^/L	0.6	0.4	0.7	0.0–1.8
Gran	10^9^/L	8.7	6.0	7.5	4.0–12.6
Lymph	%	16.4	23.9	21.6	12.0–30.0
Mon	%	5.2	5.3	6.8	2.0–9.0
Gran	%	78.4	70.8	71.6	60.0–83.0
RBC	10^12^/L	8.18	7.53	7.58	5.50–8.50
MCV	fL	74.1	73.3	72.0	62.0–72.0
MCH	pg	25.4	25.4	25.0	20.0–25.0
MCHC	g/L	343	348	341	300–380
RDW	%	12.4	12.6	12.1	11.0–15.5
PLT		201	178	192	117–460
MPV	fL	8.3	8.6	8.3	7.0–12.9
PDW	10^9^/L	16.6	17.0	16.6	
PCT	%	0.166	0.153	0.159	
Eos%	%	4.3	8.3	3.7	

**Table 2:** Biochemical parameters and CRP at baseline (T0) and post-FMT time points (T15 and T30) in the recipient.

Indicators	Unit	FMT-T0	FMT-T15	FMT-T30	Reference
GLU	mmol/L	5.82	5.75	6.06	4.11–7.95
CREA	μmol/L	167.0	119.0	121.0	44–159
UREA	mmol/L	11.9	6.8	7.1	2.5–9.6
BUN/CREA		18	14	14	
TP	g/L	68	66	66	52–82
ALB	g/L	35	33	33	23–40
GLOB	g/L	34	33	34	25–45
ALB/GLOB		1.0	1.0	1.0	
ALT	U/L	51	46	46	10–125
ALKP	U/L	44	46	50	23–212
CRP	mg/L	4.5	3.5	4.0	0.0–10.0

## Data Availability

The data presented in this study are available in the article/Supplementary Materials. Raw metagenomic sequencing data are available in the NCBI SRA under BioProject accession [PRJNA1298833]. Further inquiries can be directed to the corresponding authors.

## Supplementary Materials

The following supporting information can be downloaded at https://www.mdpi.com/article/10.3390/vetsci12090909/s1, Table S1: Relative abundance of microbial composition; Table S2: Relative abundance of KEGG pathways; Table S3: Relative abundance of COG categories.
